# Electronic epidemiological query on admission: 6 clicks for global risk assessment

**DOI:** 10.1186/1753-6561-5-S6-P34

**Published:** 2011-06-29

**Authors:** A Bispo, C Palos, M Ressureição, C Alves, J Mapril

**Affiliations:** 1 Hospital Da Luz, Portugal; 2 Siemens Medical Solutions, Lisbon, Portugal

## Introduction / objectives

Epidemiologically Important Microorganisms (EIM) reduction is a major aim of infection control. EIM are widely spread and shared between patients in several care settings. e-health, defined as intensive use of information and communication technologies, can be a major part of this strategy.

## Methods

Aiming both detection of these patients on admission and immediate implementation of procedures, Infection Control Committee (ICC) and Information Technologies team of Hospital da Luz (a high-tech, 280-bed general hospital), created an Electronic Epidemiological Query on Admission (EEQA) on the Electronic Medical Registry (EMR). EEQA comprises 6 Yes/No questions to be fulfilled by the physician in charge of admission. If at least 1 question has a positive answer (positive EEQA), it automatically generates infection control prescriptions on the EMR (specific isolation procedures for contact, airborne, droplets or Cdiff); screening cultures (nasal MRSA, rectal MRSA, VRE and MR *Acinetobacter*) or Cdiff toxin screening; activation of biohazard symbol and ICC information in order to follow-up.

## Results

For the first 50 EEQAs (starting on February), 70% were positive, resulting on measures implemented for 35 patients, with 77% global sensitivity. MRSA is the main EIM (67% sensitivity for patients who were admitted with history of health-care or long-term care stay for at least 3 days in the past 3 months).

## Conclusion

The implementation of the EEQA is an innovative approach that uses the e-health concept, allowing immediate automatic detection of high risk patients on admission, avoiding the gap of time until release of screening results. With 6 clicks, 6^6^ different protocols can be generated without any additional effort to physicians, improving quality of care.

## Disclosure of interest

None declared.

